# An Explorative Study on the Relationship between Learning Opportunities at School and at Work and Adolescents’ Mental Health

**DOI:** 10.5334/pb.516

**Published:** 2020-07-10

**Authors:** Karin Proost, Joris Van Ruysseveldt, Stef Adriaenssens, Dieter Verhaest, Dries Berings, Anja Van den Broeck

**Affiliations:** 1 KU Leuven, BE; 2 Open University of the Netherlands, NL; 3 North-West University, ZA

**Keywords:** learning opportunities, part-time job, adolescents, depression, life satisfaction

## Abstract

Based on the Job Demands-Resources model, this study examined the association of learning opportunities of adolescents at school and work with their mental health, operationalized in terms of life satisfaction and depression. Intrinsic motivation at school and at work were studied as potential mediators. Within a representative sample of adolescents (n = 474), the results supported within domain relationships in the sense that learning opportunities at school and at work were positively related to intrinsic motivation at school and at work, respectively, which in turn were related to higher levels of life satisfaction and lower levels of depression. Cross-domain relationships were not significant, except for a negative relationship between learning opportunities at work and intrinsic motivation at school, suggesting that having a good job can pull students away from school.

Even when living in relative prosperity, adolescents frequently report low levels of life satisfaction (Huebner, Drane, & Valois, 2000; Larson, 2000). At the same time, a substantial number of adolescents reports feelings of depression (Lewis, Jones, & Goodyer, 2016). This is a severe problem as feelings of dissatisfaction and depression negatively affect almost all spheres of psychosocial functioning (e.g., physical health, the quality of interpersonal relationships) and are associated with a significant impairment in multiple life domains (Frisch, 1999; Frisch et al., 2005; Furr & Funder, 1998; Lewinsohn, Redner, & Seeley, 1991).

Numerous studies have tried to understand how life satisfaction and depression develop in adolescence. These have mainly focused on family-related factors such as parental styles (Milevsky, Schlechter, Netter, & Keehn, 2007), social influences such as relationships with peers, the school teacher and the neighborhood (Oberle, Schonert-Reichl, & Zumbo, 2011; Sarkova et al., 2014), and individual difference variables such as introversion and neuroticism (Cheng & Furnham, 2003). Up to now, no study has considered the relationship between mental health of adolescents and the combination of school with part-time work. Figures, however, show that a substantial number of adolescents combine school with student work. For example, in the United States about 31% of students are engaged in paid employment (U.S. Bureau of Labor Statistics, 2006) while in Europe, 24% of 15–24 years old engage in part-time work (European Foundation for the Improvement of Living and Working Conditions, 2004).

We focus on one specific characteristic of school and part-time work, namely ‘learning opportunities’, as the primary goal of school is to offer opportunities to learn and to develop skills. Learning opportunities has also been defined as the utmost important characteristic in the work context for people’s mental health (Morrison, Cordery, Girardi, & Payne, 2005; O’Brien, 1980). We advance that looking at this characteristic both at school and at work may offer valuable new insights into adolescents’ mental health.

We add to the current literature on adolescents’ mental health in four ways. First, based on the Job Demands-Resources model (JD-R model, Bakker & Demerouti, 2007), which received support in both job and school contexts (Alarcon, Edwards, & Menke, 2011), we suggest that adolescents’ mental health is related to participation in daily activities that offer opportunities for learning and development. Second, while previous studies on adolescents’ mental health focus on one life domain or at best look at cross-over effects from student work to school (e.g., Derous & Ryan, 2008), we consider learning opportunities *in* and *across* two life domains. We argue that these domains mutually influence each other, that is from school to student work and vice-versa (Bronfenbrenner, 1992). Third, we build on Self-Determination Theory (Deci & Ryan, 1992, 2000) to suggest that intrinsic motivation at school and at work is the mediating mechanism through which learning opportunities relate to the mental health of adolescents. Finally, we consider both a positive and negative indicator of adolescents’ mental health, instead of focusing on one broad, composite measure. Doing this may help us in formulating more specific practical guidelines to stimulate mental health of adolescents (Nes et al., 2013). The conceptual model of this study is presented in Figure 1.

**Figure 1:**
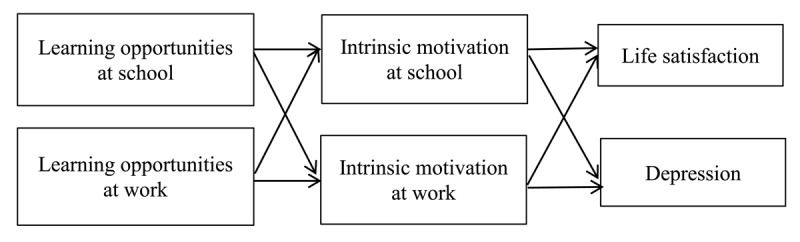
Conceptual model of the study.

## The Job Demands-Resources Model and Mental Health

The Job Demands-Resources model (Bakker & Demerouti, 2007; Bakker, Demerouti, & Sanz-Vergel, 2014) is a prominent model within the job stress literature that argues that job characteristics can be classified into job demands and job resources. Job demands are “physical, psychological, social or organizational aspects of the job that require sustained physical and/or psychological (cognitive and emotional) effort or skills and are therefore associated with certain physiological and/or psychological costs” (Bakker & Demerouti, 2007, p. 312). Examples are role conflict, an unhealthy physical environment or having an emotionally demanding job. Job resources are “physical, psychological, social or organizational aspects of the job that are either/or functional in achieving work goals, reduce job demands and the associated physiological and psychological costs and stimulate personal growth, learning and development” (p. 312). Most commonly studied examples are autonomy, social support and learning opportunities.

As implied by the definition of job resources, the JD-R model attributes a central role to learning opportunities, which are defined as opportunities to utilize and develop one’s skills (O’Brien, 1980). Several researchers have used the term ‘learning opportunities’ interchangeably with ‘skill utilization/development’ and define it as the extent to which the workplace requires the use of existing knowledge and skills but also offers opportunities to expand this knowledge and skills (Holman & Wall, 2002; Proost, Van Ruysseveldt, & van Dijke, 2012).

The JD-R model states that the availability of resources in general and the resource of learning opportunities in particular leads to enhanced well-being and life satisfaction. By having learning opportunities, people can apply their full range of expertise and even go beyond that by developing new skills and becoming even more competent (Morrison et al., 2005). As feeling effective and developing oneself is highly motivating for people, experiencing learning opportunities may lead to higher levels of mental health.

Several studies within the occupational health literature have supported this argumentation. For example, learning opportunities *at work* have been found to be one of the most important predictors of job-related well-being (Axelrod & Gavin, 1980; Kets de Vries, 2001; O’Brien 1980; Proost et al., 2012), while a lack of learning opportunities has been found to lead to job-related depression (Bracke, Pattyn, & von dem Knesebeck, 2013; Morrison et al., 2005). The relationship between learning opportunities *at school* and adolescents’ mental health, however, has been underexplored. Yet, it is long known that at all ages, people are keen to explore the world, develop and grow and engage in tasks to feel proficient and competent (White, 1959). Although a school environment should – by definition – provide learning opportunities, creating such opportunities can be “a complex and difficult process”, and should not be taken for granted (Cooner, 2010, p. 284). We advance, however, that when students see their school environment as providing many opportunities to learn, they will be more motivated and – hence – may experience improved mental health (Bakker & Demerouti, 2007; Hobfoll, 1989, 2002).

## Intrinsic Motivation as a Mediating Mechanism

The JD-R model further argues that one of the reasons why job resources are positively related to mental health is because of the intrinsic motivational role they play (Bakker & Demerouti, 2007). Intrinsic motivation is a core concept in self-determination theory (SDT, Deci & Ryan, 1992, 2000), in which it is contrasted with extrinsic motivation. While intrinsic motivation is defined as doing something because it is interesting or enjoyable, extrinsic motivation refers to doing something because it leads to a separable outcome such as money or fame or the approval of the teacher in the school context (Ryan & Deci, 2000a). Intrinsic motivation is furthermore specified as “the inherent tendency to seek out novelty and challenges, to extend and exercise one’s capacities, to explore, and to learn” (Ryan & Deci, 2000b, p. 70). Not only in childhood but throughout their lives, people want to learn, apply and develop their skills. Since learning opportunities foster this basic human tendency to learn and develop (Ryan & Deci, 2000a), we suggest that learning opportunities have an intrinsically motivating potential. Empirical evidence supports this theorizing: Learning opportunities have been shown to lead to more pleasure and fun in the task (Harter, 1978; White, 1959) and students who can deploy and develop their skills, reported to experience higher levels of intrinsic motivation in the task (Ryan & Deci, 2000b).

SDT further argues that doing something because it is fun and exciting, i.e., that is intrinsically motivating, increases general well-being (Deci & Ryan, 2000). Many empirical studies support this assumption. For example, Burton, Lydon, D’Alessandro, and Koestner (2006) found significant positive relationships over time between intrinsic motivation and positive affect among elementary school children. They further showed in an experimental study among university students that manipulating intrinsic motivation led to higher levels of life satisfaction. The relationship between intrinsic academic motivation and depression was shown by Huang, Lv, and Wu (2016) among undergraduate students.

Building on this literature, we formulated the following hypotheses:

*Hypothesis 1*: Intrinsic motivation at *school* (partially) mediates the positive relationship of learning opportunities at *school* with life satisfaction and the negative relationship with depression.

*Hypothesis 2*: Intrinsic motivation at *work* (partially) mediates the positive relationship of learning opportunities at *work* with life satisfaction and the negative relationship with depression.

## Cross-Domain Relationships Between School and Student Work

Research traditionally looked at school and student work as distinct domains. The current literature, however, moved away from this conceptualization and considers both domains as interconnected. This idea is grounded in ecological systems theory, which reasons that people engage in a web of interrelated contexts in which they adopt different roles (Bronfenbrenner, 1992). Ford, Heinen, and Langkamer (2007) refer to ‘cross-domain influences’, which can be defined as the extent to which characteristics of one domain influence the outcomes in the other domain.

Several studies focused on influences from the work domain to the school domain and showed a rather pessimistic picture (see Neyt, Omey, Verhaest, & Baert, 2019 for a literature review). For example, Steinberg, Fegley, and Dornbusch (1993) showed that students who engaged in paid work more than 20 hours per week became more disengaged from school and Warren and Cataldi (2006) showed that this trend is stable among students since the late 1960s. Similarly, Lens, Lacante, Vansteenkiste, and Herrera (2005) showed a negative relationship between time spent in student work and study motivation, study attitude, and academic achievement. McCoy and Smyth (2007) showed that part-time employment leads to underperformance and increased dropout among students in secondary education, even when controlled for possible selection effects by using propensity score matching methods. Some studies, however, showed that students who worked less than 20 hours per week, profited from this student work in terms of better school performance, higher likelihood of going to the university and lower risk of dropping out of school (Lee & Staff, 2007; Stern & Briggs, 2001). These contradicting findings suggest that the quantity of the time spent in work matters.

Other studies showed that besides (and even more than) quantity, the quality of the job is important (Staff & Schulenberg, 2010). These studies showed a more optimistic picture of how paid work can have beneficial effects for students. For example, Mortimer (2003) showed that student work is conducive to academic (i.e., educational achievement) and non-academic outcomes (i.e., occupational identity, goal setting) to the extent that the job encourages decision making, career exploration, confidence-building, and competence. Also, student jobs that are relevant for the study and are performed in an autonomous way have been found to benefit students’ academic performance (Derous & Ryan, 2008).

In general, these studies show that engagement in one activity and/or domain may have consequences for another activity and/or domain. On the one hand, people may lose resources (e.g., energy, time) when they perform multiple tasks, especially in the case of role conflict. For example, when student work is time consuming, adolescents have fewer resources to spend at school and at the same time need to invest more of their resources into the work role in order not to lose their work status (Neyt et al., 2019). This actual loss of resources leads to underperformance and distress (Grandey & Cropanzano, 1999) and may explain the negative results reported above. On the other hand, when resources (e.g., learning opportunities) can be gained in one domain, this can help people to cope with a lack of resources in another domain, or even gain resources in that domain, resulting in increased mental health (Hobfoll, 2002). For example, when adolescents can use and expand their skills in one domain through the learning opportunities offered, this may not only increase their intrinsic motivation in that and the other domain but also their general well-being. The reason for this is that the context-specific resource of learning opportunities may create more general resources such as energy and self-esteem, which can be used in other domains as well. This bigger arsenal of resources may help students than to cope with a lack of resources in the other domain and even build additional resources in this other domain, leading to increased intrinsic motivation and thus improved mental health (Baumeister, Campbell, Krueger, & Vohs, 2003; Orth, Robins, & Roberts, 2008).

Although the discussed empirical evidence only supports a positive spillover from work to school, theoretically this positive spillover may just as much work in the other direction. For example, students can gather resources such as knowledge and skills but also self-esteem and energy at school which can help them to experience the student job as more challenging and pleasant. Based on this literature, we suggested that learning opportunities in one domain relate positively to intrinsic motivation in the other domain and hence relate positively to mental health through this cross-over effect. Specifically, we formulated the following mediation hypotheses:

*Hypothesis 3*: Intrinsic motivation at *work* (partially) mediates the positive relationship of learning opportunities at *school* with life satisfaction and the negative relationship with depression.

*Hypothesis 4*: Intrinsic motivation at *school* (partially) mediates the positive relationship of learning opportunities at *work* with life satisfaction and the negative relationship with depression.

## Method

### Participants and Procedure

Data for this study were collected as part of a larger survey on student employment in the Dutch speaking part of Belgium. In order to guarantee representativeness, we used an indirect sampling technique with probability proportional to size weights. The sampling frame consisted of all the schools organizing second and third grade secondary education in the Flemish Region, in general, technical and vocational formation (excluding students in dual learning trajectories and in Arts education). We obtained this list from the Flemish Education Administration. In order to reduce clustering, we chose to construct elementary grade-formation units of student groups of one formation and one grade (so e.g., the second grade technical students of school X). The frame contained 2711 of these elementary grade-formation units in the Flemish Region. Initially, 60 units were representatively sampled in such a way that the chance for a unit to be selected depended on the number of students in the unit (this is the probability proportional to size weighting). In this way, each student had an equal chance to be selected. Schools that dropped out were replaced by another school, matched in terms of region, school net, and school type. In the end, 34 schools participated in the study.

Data collection occurred in two ways. In some schools, a university student, as part of the master thesis, collected the data during regular class hours. In other schools, questionnaires were administered by a teacher. In both cases, a strict protocol was followed in order to guarantee standardization of administration (see Adriaenssens, Verhaest, Van den Broeck, Proost, & Berings, 2014, for a detailed discussion of the sampling method). We clearly defined student work as part-time work that students got paid for in order to exclude unpaid traineeships or volunteering.

For the variables that were used in this study, 474 completed questionnaires were gathered from students that worked on a regular basis during the start of the new school year. 57% of the respondents were female, 17% of the students followed general secondary education, 46% followed technical education and 37% followed vocational education. On average students worked 9.82 hours per week (*SD* = 15.12) and worked one day in the week (49%), mostly on Saturdays (76%). The average age of the respondents was 17.79 years (*SD* = 1.27). Table 1 gives an overview of the types of jobs students engaged in. The majority of the students worked in commercial and administrative jobs, followed by catering, tourism, recreation, transport and logistics.

**Table 1:** Overview of types of jobs students engaged in.

Occupational Field		Examples

Commercial and administrative	34.6%	Cashier (9.1%), general shop assistant (7.2%), stock clerk (5.9%), clothes shop assistant (4.4%)
Catering, tourism, recreation, transport and logistics	32.1%	Barkeeper/waitress (10.8%), busboy (6.5%), kitchen employee (3.0%), fast-food employee (2.7%)
Environmental and agricultural	9.5%	Dishwasher (4.9%), carwash attendant (0.6%), industrial cleaner (0.6%)
Techncal	8.6%	Bakery operator (1.3%), Handyman (1.1%), navvy (0.4%)
Caring and social work	5.5%	Babysitter (2.7%), caring assistant (1.9%), barber assistant (0.6%)
Education	5.5%	Sport instructor (4.4%), dance teacher (0.8%)
Other fields	1.7%	Security guard (0.4%)
Unknown/missing	2.5%	

### Measures

#### Learning opportunities at school and at work

The Leiden Quality of Work Questionnaire (LAKS, van der Doef & Maes, 1999) was used to measure learning opportunities at work and a commensurate measure was developed to measure learning opportunities at school. Both scales were measured with 4 items. Sample items are ‘My studies require that I learn new things’ and ‘My work requires that I learn new things’. Items were answered on a Likert-type scale, ranging from 1 (*= totally disagree*) to 5 (*= totally agree*). Cronbach’s alphas for learning opportunities at school and work were .76 and .83, respectively.

#### Intrinsic motivation at school and at work

Commensurate measures were also used to measure intrinsic motivation at school and at work. Both scales were measured with 2 items, adopted from the Questionnaire on the Experience and Evaluation of Work (QEEW, Veldhoven & Meijman, 1994). Sample items are ‘I enjoy school’ and ‘I enjoy work’. Items were answered on a scale from 1 (= *totally disagree*) to 5 (= *totally agree*). Cronbach’s alphas for the scales on intrinsic motivation at school and work were .77 and .96, respectively. We believe these items accurately tap into intrinsic motivation (i.e., “doing something because it is interesting or enjoyable”, Ryan & Deci, 2000a, p. 55) as they question whether students enjoy school and work and find pleasure in doing it.

#### Life satisfaction

Life satisfaction was measured with the Satisfaction with Life Scale (Diener, Emmons, Larsen, & Griffin, 1985). This scale has been extensively studied and showed adequate construct validity and internal consistency and is suited for use with adolescents (Diener et al., 1985; Pavot & Diener, 1993). A sample item is: ‘In most ways my life is close to my ideal’. The scale consisted of 5 items which were answered on a Likert-type scale from 1 (= *totally disagree*) to 5 (= *totally agree*). Cronbach’s alpha for this scale was .81.

#### Depression

Depression was measured with the Center for Epidemiologic Studies Depression Scale (Radloff, 1977). This is a sound psychometric instrument to detect depression (Roberts, Andrews, Lewinsohn, & Hops, 1990). Several short versions have been developed and we used the 12-item version, validated by Roberts and Sobhan in a sample of adolescents (1992). A sample item is ‘During the past week, I felt depressed’. Items were measured on a Likert-type scale from 1 (= *rarely or none of the time, i.e., less than 1 day*) to 4 (= *most or all of the time, 5–7 days*). Cronbach’s alpha of this scale was .84, showing adequate internal consistency.

In order to verify the construct validity of these measures, we included all independent and dependent variables in an exploratory factor analysis with oblique rotation (i.e., promax with Kaiser normalization). Rather than showing the expected six factors (i.e., learning opportunities at school/work, intrinsic motivation at school/work, life satisfaction and depression), based on the criterion of eigenvalue > 1 and the scree plot, this analysis revealed a solution with seven factors. The items measuring depression loaded on two instead of one factor. Specifically, the positively worded items ‘felt just a good as other people’, ‘felt hopeful about the future’, ‘was happy’ and ‘enjoyed life’ loaded on a separate factor. To further establish the factor structure of our data, we subsequently conducted two confirmatory factor analyses with maximum likelihood estimation. A 6-factor solution showed a moderate fit to the data, *X²* = 914.51, *p* = .00, *RMSEA* = .06, *CFI* = .90, *SRMR* = 0.10, but only after allowing covariances between error terms for the positively worded depression items. This suggests that these items may load on a separate latent factor. The 7-factor solution with two latent factors for depression, one for the negatively worded items and one for the positively worded items, held a better fit to the data, *X²* = 878.06, Δχ² = 36.45, Δdf = 4, *p* < .01, *RMSEA* = .05, *CFI* = .90, *SRMR* = 0.09. However, since the literature does not evidence two factors for depression and since the Cronbach’s alpha of the depression scale including both positively and negatively worded items indicated good internal consistency (i.e., α = .84), we decided to keep these items together in one scale on depression. We also did not want to opt for removing the positively worded items from the scale as it lowered the internal consistency to .81.

#### Control variables

To control for the heterogeneity in the dataset, we included age, gender (0 = *male*; 1 = *female*) and two dummies for type of education (1 = *general secondary education*, 0 = *other*; 1 = *technical education*, 0 = *other*) in the analyses. Previous research has shown that older students and students in vocational education (followed by students in technical and general secondary education) participate more in student work (Adriaenssens et al., 2014) and that women report higher rates of depression than men (Jack, 1991). We also controlled for time spent in the job (“How many hours do you work per week?”) since we know from the literature review that spending more time in the job may lead to several negative outcomes (Lens et al., 2005; Steinberg et al., 1993; Warren & Cataldi, 2006).

### Analyses

To test the hypothesized model, we used Structural Equation Modeling (SEM; STATA 15.0) with maximum-likelihood estimation. We believe SEM is the most appropriate technique as it allows to test these more complicated models in one analysis and provides model fit information. It also allows to control for correlations between the dependent variables (MacKinnon, 2008).

In the following, we make a distinction between mediating effects and indirect effects, both falling under the concept of ‘intervening effects’ (Mathieu & Taylor, 2006). Mediation occurs when a significant direct relationship between the independent and dependent variable is explained by a third variable, the mediator. In the absence of this direct relationship, two variables can still be linked to each other through a third variable. In line with this more modern approach, we are also interested in a possible chain of events where the independent and the dependent variable are indirectly related through significant relationships of both of them with a ‘linking’ variable. Rather than speaking of mediation, this is called an ‘indirect effect’ and the assessment of it does not require a significant direct relationship between the independent and dependent variable (Hayes, 2009; Preacher & Hayes, 2008). We respect this difference in the rest of this text and use the term ‘intervening effects’ as the umbrella term. In order to test the hypotheses, the fit of the hypothesized model was compared to the fit of three alternative models with intervening effects (learning opportunities at school to all outcomes; learning opportunities at work to all outcomes; both learning opportunities to all outcomes).

## Results

The descriptive statistics and correlations between the variables in this study are presented in Table 2. Learning opportunities at school were positively related to intrinsic motivation both at school and at work as well as to life satisfaction but not to depression. Learning opportunities at work were positively related to intrinsic motivation at work and life satisfaction but also not to depression. Intrinsic motivation (at school and at work) was positively related to life satisfaction and negatively related to depression.

**Table 2:** Descriptive statistics and intercorrelations between the variables in this study.

	*M*	*SD*	2	3	4	5	6	7	8	9	10	11

1. Age	17.79	1.27	.04	–.18**	.12**	.06	.07	–.01	.05	.01	–.09	.13**
2. Gender	.57	.50		.13**	.09*	–.17**	.07	–.17**	.10*	.04	–.08	.07
3. General education	.17	.38			–.42**	–.07	.03	–.05	.05	–.03	.01	–.02
4. Technical education	.46	.50				–.11*	.12**	–.14**	.03	.05	.01	–.01
5. Time spent in the job	9.73	11.05					–.03	.08	–.03	.01	.07	–.02
6. LO at school	3.39	.80						.10*	.30**	.09*	.16**	–.03
7. LO at work	2.99	.93							–.09	.33**	.10*	.03
8. Intrinsic motivation school	3.05	1.03								.14**	.21**	–.23**
9. Intrinsic motivation work	3.40	1.14									.26**	–.15**
10. Life satisfaction	3.42	.76										–.40**
11. Depression	1.90	.54										

**: p < .01; *: p < .05; +: p < .07. LO = learning opportunities.

**Figure 2:**
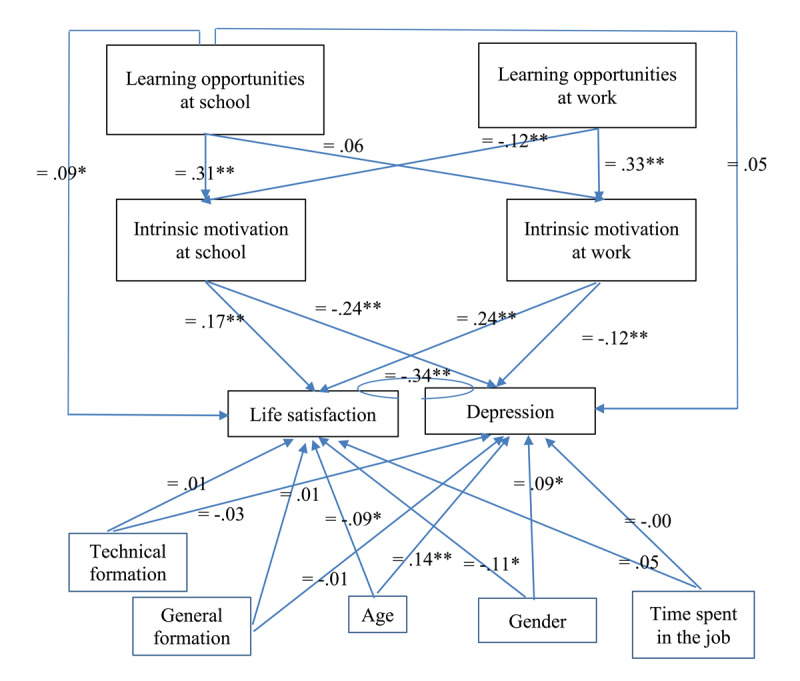
Results of SEM analysis (Model B).

The hypothesized model (Model A, see Figure 1) fitted the data well (χ² = 35.85, *RMSEA* = .05, *CFI* = .93, *SRMR* = .03). The fit of this model was then compared to three alternative models. The model including direct paths from learning opportunities at school to the dependent variables showed a significant better fit to the data (Model B, χ² = 28.26, Δχ² = 7.59, Δdf = 2, *p* < .05). Also the model including direct paths from learning opportunities at work to the dependent variables led to a significant improvement in fit (Model C, χ² = 31.79, Δχ² = 4.06, Δdf = 2, *p* < .05) but these direct paths did not reach significance (i.e., *β* = .02, *p* = .60 for life satisfaction and *β* = .08, *p* = .09 for depression). Finally, the model including all direct relationships between the two independent variables and both dependent variables showed an improvement in fit compared to model A (χ² = 25.35, Δχ² = 10.50, Δdf = 4, *p* < .01) but not compared to Model B (Δχ² = 2.91, Δdf = 2, *ns*). For reasons of parsimony, we report and interpret findings from Model B (see Figure 2).

Hypotheses 1 and 2 suggested a mediating role of intrinsic motivation within the respective domain on the relationships between learning opportunities and the outcome variables. Concerning the school context, the direct path from learning opportunities at school to intrinsic motivation at school was significantly positive (β = .31, *p* = .00). Further, intrinsic motivation at school was significantly positively related to life satisfaction (β = .17, *p* = .00). Since also the correlation between learning opportunities at school and life satisfaction was significant, one can speak of mediation and more specifically partial mediation since also the direct path from learning opportunities at school to life satisfaction was still significantly positive (β = .09, *p* = .05). Intrinsic motivation at school was also negatively related to depression (β = –.24, *p* = .00). However, since the correlation between learning opportunities at school and depression was not significant, we speak of an indirect effect between learning opportunities at school and depression.

The same results were found with respect to the work context. The direct path from learning opportunities at work to intrinsic motivation at work was significantly positive (β = .33, *p* = .00) and intrinsic motivation at work related positively to life satisfaction (β = .24, *p* = .00). Given that the direct relationship between learning opportunities at work and life satisfaction turned insignificant after controlling for intrinsic motivation at work, we can speak of full mediation. The path to depression was significantly negative (β = –.12, *p* = .01), showing a significant indirect effect.

Concerning hypothesis 3 and 4, suggesting cross-domain relationships, the path from learning opportunities at school to intrinsic motivation at work was not significant (β = .06, *p* = .20). The direct path from learning opportunities at work to intrinsic motivation at school was significant but in the opposite direction as hypothesized (β = –.12, *p* = .01). As such, hypotheses 3 and 4 were not confirmed by the data.

With respect to the demographic variables, the older group of adolescents experienced lower levels of life satisfaction (β = .–.09, *p* = .03) and higher levels of depression (β = .14, *p* = .00). Also female respondents reported lower levels of life satisfaction (β = –.11, *p* = .01) and higher levels of depression (β = .09, *p* = .05). Other relationships were not significant.

## Discussion

This study aimed to investigate whether and how learning opportunities at school and at work influence life satisfaction and depression of adolescents. This study is the first to consider learning opportunities as a way to increase mental health of adolescents. Based on the JD-R model (Bakker & Demerouti, 2007; Bakker et al., 2014) and SDT (Deci & Ryan, 1992; 2000), intrinsic motivation at school and at work was suggested as an explanatory mechanism. This study adds to the literature on adolescents’ mental health by integrating ideas from the occupational health literature with ideas from the educational literature, and thus allows to formulate new insights into the promotion of mental health of adolescents.

### Main Findings and Theoretical Implications

We concentrated on learning opportunities at school and work as a way to increase students intrinsic motivation in each domain and consequently their mental health. We focused on the meaning of part-time work as a way to increase the mental health of adolescents. Based on the JD-R model (Bakker & Demerouti, 2007; Bakker et al., 2014), we suggested that learning opportunities have important motivational implications and as such the potential to increase mental health. We predicted relationships both within and across domains on life satisfaction and depression, through intrinsic motivation. The results confirm the within domain hypotheses in the sense that learning opportunities at school and at work were positively related to life satisfaction and depression via intrinsic motivation at school and at work, respectively. This suggests, in line with SDT (Deci & Ryan, 1992, 2000), that learning opportunities may indeed have an important motivational potential and as such increase mental health.

No support is found for the predicted cross-domain relationships. This is surprising as studies have shown that gaining resources in one domain can help people to cope with demands or gain additional resources in other domains. This idea has also received support in the literature on combining school with student work, showing that the combination of school with a high quality job leads to improved academic performance (Derous & Ryan, 2008; Mortimer, 2003; Shanahan & Flaherty, 2001). A possible explanation for the lack of significance in our sample may be the limited amount of time that students spend in student work. The students in this sample mainly worked one day a week in their student work, mostly on Saturdays. The fact that both activities were clearly separated in time may explain why no cross-domain influences were found.

Another possible explanation may be the limited resemblance between the skills that were developed in the studies versus the job. Derous and Ryan (2008) suggested that positive cross-domain effects may only be expected when the activities in one domain are perceived as relevant for the other domain. We studied relatively young adolescents who were involved in student jobs for which typically no education was requested (e.g., helping in a restaurant, see Table 1). As the skills necessary for these jobs differ significantly from the skills at school, feelings of competence and intrinsic motivation in one domain may not cross-over to the other domain. However, for students in vocational education, the boundaries between school and work may be less straightforward due to e.g., traineeships. Also the kind of jobs they engage in outside of school, may be more strongly linked to the skills they learn at school (see Adriaenssens et al., 2014). As such, for this specific group of students, stronger cross-domain relationships could be expected. We therefore looked at the cross-domain relationships separately for each educational type and found that although the relationship between learning opportunities at school and intrinsic motivation at work was not significant in the total sample, nor in the group of students in general and technical education, this relationship became significant in the group of students in vocational education (i.e., *r* = –.04 for general education, *r* = –.01 for technical education, *r* = .20, *p* < .01 for vocational education). The skills learned at school may be more easily transferrable for this group of students to the work context, hence this significant relationship between what they learn at school and how motivated they are in their part-time job. Future studies on the combination of work and study could pay more attention to specific learning trajectories that make the transfer from learning to work more easy and may stimulate students’ intrinsic motivation across domains.

The negative relationship between learning opportunities at work and intrinsic motivation at school was remarkable. Apparently, when students find a job in which they can develop their skills and consequently become intrinsically motivated for the job, this relates negatively to their motivation at school. Although future studies are needed to settle on the causality of this relationship, this suggests that a good job may stimulate a pull mechanism by attracting students towards it but at the same time also a push mechanism by pushing students away from school. This may especially be the case for students who already feel bored at school and who, through finding a motivating job, feel strengthened in their idea that they do not belong at school (see McNeal, 1997; Warren & Cataldi, 2006).

### Limitations

The sample for this study was selected in a representative way, guaranteeing the population validity of our study. However, there may be some limitations to the data collection as we used a cross-sectional design and self-report measures. As mentioned above, this way of collecting data (i.e., cross-sectional) implies that we cannot claim causality. We followed, however, some suggestions of Van der Stede (2014) to infer causality from cross-sectional studies. First, the directions of the relationships proposed in our model were substantiated by a strong theoretical framework (i.e., JD-R model, Bakker & Demerouti, 2007; Bakker et al., 2014). Also numerous empirical studies in this domain have supported longitudinal effects of job characteristics on well-being (e.g., Hakanen, Schaufeli, & Ahola, 2008). Second, we controlled for some possible confounding variables that could offer alternative explanations for the results obtained. For example, students in vocational education may observe less learning opportunities at school than students in general education. Other studies suggest a relationship between type of education and life satisfaction (Salmela-Aro & Tuominen-Soini, 2010; Wouters, De Fraine, Colpin, Van Damme, & Verschueren, 2012), as well as to depression (Bjelland et al., 2008). As such, type of education may relate to both learning opportunities at school and indicators of mental health, leading to a spurious correlation between learning opportunities at school and mental health. By controlling for this variable, we excluded this alternative explanation. However, the results remain explorative and should be replicated by other methodologies.

Second, self-reports are often accused from leading to common method bias although this criticism to self-reports is recently placed into question by several authors (Chan, 2009; Conway & Lance, 2010). Moreover, we believe that self-reports were the most relevant measurement method in this study as we were basically interested in the subjective perceptions of the adolescents’ themselves about the learning opportunities in their study and job, their own experiences of intrinsic motivation in the study and in the job, as well as their own perceptions of well-being. Previous studies have shown that subjective perceptions of job characteristics are related to the objective job characteristics but that especially the first are relevant with respect to psychosocial outcome variables (Melamed, Ben-Avi, Luz, & Green, 1995). Another paper in Science by Oswald and Wu (2010) showed that measures of subjective well-being provide genuine information about the objective well-being of people. Based on this literature, we are confident that self-reports were the most appropriate measurement methods for this study.

Another limitation of this study is that we focused solely on learning opportunities, which is only one possible resource within the JD-R model. Other resources that may play an important role are supervisory/teacher coaching, performance feedback and autonomy (Bakker & Demerouti, 2007). Also, to measure learning opportunities at school and at work, we used a well-validated scale in the domain of work-related learning (i.e., Leiden Quality of Work Questionnaire, Van der Doef & Maes, 1999). This measure is often used but is rather general and might not have captured the quality of the learning experience. This may also explain the lack of significance of the cross-domain relationships. Further studies could try to use different measures that capture whether the school environment is really perceived as a challenging, powerful learning environment or not (see e.g., Gentry & Springer, 2002; Young, Williamson, & Egan, 2016) or to use measures that capture more strongly the learning potential of the school/work environment (see Nikolova, Van Ruysseveldt, De Witte, & Syroit, 2014).

Finally, we focused on two life domains of adolescents, namely school and student work. Adolescents, however, also participate in other activities such as extra-curricular activities offered by school (e.g., school radio stations, school newspaper), or other activities outside of school such as sports, youth associations and (electronic) networking with friends. These activities may be considered in future research as they may have an impact on academic performance, attitudes and aspirations (Darling, Caldwell, & Smith, 2005) as well as on adolescents well-being (Valkenburg, Peter, & Schouten, 2006).

### Practical Implications

Research indicates that students often feel bored at school (Daschmann, Goetz, & Stupnisky, 2011; Larson & Richards, 1991). The results of the present study suggest that this problem can be overcome by offering students the opportunity to develop and deploy their skills at school. In this way, school activities will be experienced as fun and challenging. Also the problem of low levels of life satisfaction and frequently reported feelings of depression may indirectly be tackled in this way. By intrinsically motivating students at school, life satisfaction increases and feelings of depression decrease.

Should students also be stimulated to combine their study with a student job? The present study suggests that motivation at school cannot benefit from doing so. However, students’ well-being can be improved by looking for another domain in which one can deploy one’s skills. As the results of this study showed, by offering students the opportunity to learn at work, they feel intrinsically motivated at work and thus are more satisfied with their lives and experience lower levels of depression. In this study, we only focused on student work but possibly, other domains can also offer this opportunity to learn and thus experience intrinsic motivation and as such increase the well-being of adolescents.

## Conclusion

The main aim of this study was to examine the relationship between learning opportunities at school and at work and adolescents’ mental health. Relationships were examined within and across both domains and intrinsic motivation was considered as a possible mediator. The results supported the within-domain relationships but not the expected cross-domain relationships between learning opportunities and mental health.

## References

[1] Adriaenssens, S., Verhaest, D., Van den Broeck, A., Proost, K., & Berings, D. (2014). De arbeidsparticipatie van Vlaamse scholieren. Tijdschrift voor Arbeidsvraagstukken, 30(3), 281–301. https://www.tijdschriftvoorarbeidsvraagstukken.nl/inhoud/tijdschrift_artikel/TA-30-3-281/De-arbeidsparticipatie-van-Vlaamse-scholieren DOI: 10.5553/TvA/016922162014030003006

[2] Alarcon, G. M., Edwards, J. M., & Menke, L. E. (2011). Student burnout and engagement: A test of the conservation of resources theory. The Journal of Psychology: Interdisciplinary and Applied, 145(3), 211–227. DOI: 10.1080/00223980.2011.555432 21560805

[3] Axelrod, W. L., & Gavin, J. F. (1980). Stress and strain in blue-collar and white-collar management staff. Journal of Vocational Behavior, 17(1), 41–49. DOI: 10.1016/0001-8791(80)90013-5

[4] Bakker, A. B., & Demerouti, E. (2007). The job demands-resources model: State of the art. Journal of Managerial Psychology, 22(3), 309–328. DOI: 10.1108/02683940710733115

[5] Bakker, A. B., Demerouti, E., & Sanz-Vergel, A. I. (2014). Burnout and work engagement: The JD-R approach. Annual Review of Organizational Psychology and Organizational Behavior, 1, 389–411. DOI: 10.1146/annurev-orgpsych-031413-091235

[6] Baumeister, R. F., Campbell, J. D., Krueger, J. I., & Vohs, K. D. (2003). Does high self-esteem cause better performance, interpersonal success, happiness, or healthier lifestyles? Psychological Science in the Public Interest, 4(1), 1–44. DOI: 10.1111/1529-1006.01431 26151640

[7] Bjelland, I., Krokstad, S., Mykletun, A., Dahl, A. A., Tell, G. S., & Tambs, K. (2008). Does a higher educational level protect against anxiety and depression? The HUNT study. Social Science & Medicine, 66, 1334–1345. DOI: 10.1016/j.socscimed.2007.12.019 18234406

[8] Bracke, P., Pattyn, E., & von dem Knesebeck, O. (2013). Overeducation and depressive symptoms: Diminishing mental health returns to education. Sociology of Health & Illness, 35(8), 1242–1259. DOI: 10.1111/1467-9566.12039 23909439

[9] Bronfenbrenner, U. (1992). Ecological systems theory In V. Ross (Ed.), Six Theories of Child Development: Revised Formulations and Current Issues (pp. 187–249). London, England: Jessica Kingsley Publishers.

[10] Burton, K. D., Lydon, J. E., D’Alessandro, D. U., & Koestner, R. (2006). The differential effects of intrinsic and identified motivation on well-being and performance: Prospective, experimental, and implicit approaches to self-determination theory. Journal of Personality and Social Psychology, 91(4), 750–762. DOI: 10.1037/0022-3514.91.4.750 17014297

[11] Chan, D. (2009). So why ask me? Are self-report data really that bad? In C. E. Lance & R. J. Vandenberg (Eds.), Statistical and methodological myths and urban legends: Doctrine, verity and fable in the organizational and social sciences (pp. 311–338). New York, NY: Routledge.

[12] Cheng, H., & Furnham, A. (2003). Personality, self-esteem, and demographic predictions of happiness and depression. Personality and Individual Differences, 34(6), 921–942. DOI: 10.1016/S0191-8869(02)00078-8

[13] Conway, J. M., & Lance, C. E. (2010). What reviewers should expect from authors regarding common method bias in organizational research. Journal of Business and Psychology, 25, 325–334. DOI: 10.1007/s10869-010-9181-6

[14] Cooner, T. S. (2010). Creating opportunities for students in large cohorts to reflect in and on practice: Lessons learnt from a formative evaluation of students’ experiences a technology-enhanced blended learning design. British Journal of Educational Technology, 41, 271–286. DOI: 10.1111/j.1467-8535.2009.00933.x

[15] Darling, N., Caldwell, L. L., & Smith, R. (2005). Participation in school-based extracurricular activities and adolescent adjustment. Journal of Leisure Research, 37(1), 51–76. http://search.proquest.com/openview/074b720851b5738c773850e03158bfb7/1?pq-origsite=gscholar&cbl=34631 DOI: 10.1080/00222216.2005.11950040

[16] Daschmann, E. C., Goetz, T., & Stupnisky, R. H. (2011). Testing the predictors of boredom at school: Development and validation of the precursors to boredom scales. British Journal of Educational Psychology, 81(3), 421–440. DOI: 10.1348/000709910X526038 21770913

[17] Deci, E. L., & Ryan, R. M. (1992). The initiation and regulation of intrinsically motivated learning and achievement In A. K. Boggiano & T. S. Pittman (Eds.), Achievement and motivation: A social developmental perspective (pp. 9–36). New York, NY: Cambridge University Press.

[18] Deci, E. L., & Ryan, R. M. (2000). The “What” and “Why” of goal pursuits: Human needs and the self-determination of behavior. Psychological Inquiry: An International Journal for the Advancement of Psychological Theory, 11(4), 227–268. DOI: 10.1207/S15327965PLI1104_01

[19] Derous, E., & Ryan, A. M. (2008). When earning is beneficial for learning: The relation of employment and leisure activities to academic outcomes. Journal of Vocational Behavior, 73(1), 118–131. DOI: 10.1016/j.jvb.2008.02.003

[20] Diener, E., Emmons, R. A., Larsen, R. J., & Griffin, S. (1985). The satisfaction with life scale. Journal of Personality Assessment, 49(1), 71–75. DOI: 10.1207/s15327752jpa4901_13 16367493

[21] European Foundation for the Improvement of Living and Working Conditions. (2004). Part-time work in europe. http://www.eurofound.europa.eu/ewco/reports/TN0403TR01/TN0403TR01.htm

[22] Ford, M. T., Heinen, B. A., & Langkamer, K. L. (2007). Work and family satisfaction and conflict: A Meta-analysis of cross-domain relations. Journal of Applied Psychology, 92(1), 57–80. DOI: 10.1037/0021-9010.92.1.57 17227151

[23] Frisch, M. B. (1999). Quality of Life Assessment/Intervention and the Quality of Life Inventory (QQLI) In M. R. Maruish (Ed.), The use of psychological testing for treatment planning and outcome assessment (2nd ed., pp. 1227–1331). Hillsdale, NJ: Lawrence Erlbaum.

[24] Frisch, M., Clark, M. P., Rouse, S. V., Rudd, M. D., Paweleck, J., Greenstone, A., & Kopplin, D. (2005). Predictive and treatment validity of life satisfaction and the quality of life inventory. Assessment, 12(1), 66–78. DOI: 10.1177/1073191104268006 15695744

[25] Furr, R. M., & Funder, D. (1998). A multimodal analysis of personal negativity. Journal of Personality and Social Psychology, 74(6), 1580–1591. DOI: 10.1037/0022-3514.74.6.1580 9654761

[26] Gentry, M., & Springer, P. M. (2002). Secondary student perceptions of their class activities regarding meaningfulness, challenge, choice, and appeal: An initial validation study. The Journal of Secondary Gifted Education, 13, 192–204. DOI: 10.4219/jsge-2002-381

[27] Grandey, A. A., & Cropanzano, R. (1999). The conservation of resources model applied to work-family conflict and strain. Journal of Vocational Behavior, 54(2), 350–370. DOI: 10.1006/jvbe.1998.1666

[28] Hakanen, J. J., Schaufeli, W. B., & Ahola, K. (2008). The Job Demands-Resources model: A three-year cross-lagged study of burnout, depression, commitment, and work engagement. Work & Stress, 22, 224–241. DOI: 10.1080/02678370802379432

[29] Harter, S. (1978). Pleasure derived from challenge and the effects of receiving grades on children’s difficulty level choices. Child Development, 49(3), 788–799. DOI: 10.2307/1128249

[30] Hayes, A. F. (2009). Beyond Baron and Kenny: Statistical mediation analysis in the new millennium. Communication Monographs, 76, 408–420. DOI: 10.1080/03637750903310360

[31] Hobfoll, S. E. (1989). Conservation of resources: A new attempt at conceptualizing stress. American Psychologist, 44(3), 513–524. DOI: 10.1037/0003-066X.44.3.513 2648906

[32] Hobfoll, S. E. (2002). Social and psychological resources and adaptation. Review of General Psychology, 6(4), 307–324. DOI: 10.1037/1089-2680.6.4.307

[33] Holman, D. J., & Wall, T. D. (2002). Work characteristics, learning-related outcomes, and strain: A test of competing direct effects, mediated, and moderated models. Journal of Occupational Health Psychology, 7(4), 283–301. DOI: 10.1037/1076-8998.7.4.283 12396063

[34] Huang, Y., Lv, W., & Wu, J. (2016). Relationship between intrinsic motivation and undergraduate students’ depression and stress: The moderating effect of interpersonal conflict. Psychological Reports, 119, 527–538. DOI: 10.1177/0033294116661512 27488914

[35] Jack, D. C. (1991). Silencing the self: Women and depression. Cambridge, MA: Harvard University Press.

[36] Kets de Vries, M. F. R. (2001). Creating authentizotic organizations: Well-functioning individuals in vibrant companies. Human Relations, 54(1), 101–111. DOI: 10.1177/0018726701541013

[37] Larson, R. W. (2000). Toward a psychology of positive youth development. American Psychologist, 55(1), 170–183. DOI: 10.1037/0003-066X.55.1.170 11392861

[38] Larson, R. W., & Richards, M. H. (1991). Boredom in the middle school years: Blaming schools versus blaming students. American Journal of Education, 99(4), 418–43. DOI: 10.1086/443992

[39] Lee, J. C., & Staff, J. (2007). When work matters: The varying impact of work intensity on high school dropout. Sociology of Education, 80(2), 158–178. DOI: 10.1177/003804070708000204

[40] Lens, W., Lacante, M., Vansteenkiste, M., & Herrera, D. (2005). Study persistence and academic achievement as a function of the type of competing tendencies. European Journal of Psychology of Education, 20(3), 275–287. DOI: 10.1007/BF03173557

[41] Lewinsohn, P. M., Redner, J., & Seeley, J. R. (1991). The relationship between life satisfaction and psychosocial variables: New perspectives In F. Strack, M. Argyle, & N. Schwarz (Eds.), Subjective Well-being (pp. 193–212). New York, NY: Plenum Press.

[42] MacKinnon, D. (2008). Introduction to statistical mediation analysis. New York, NY: Lawrence Erlbaum Associates.

[43] Mathieu, J. E., & Taylor, S. R. (2006). Clarifying conditions and decision points for mediational type inferences in organizational behavior. Journal of Organizational Behavior: The International Journal of Industrial, Occupational, and Organizational Psychology and Behavior, 27, 1031–1056. DOI: 10.1002/job.406

[44] McCoy, S., & Smyth, E. (2007). So much to do, so little time. Part-time employment among secondary students in Ireland. Work, Employment and Society, 21(2), 227–246. DOI: 10.1177/0950017007076630

[45] McNeal, R. B., Jr. (1997). Are students being pulled out of high school? The effect of adolescent employment on dropping out. Sociology of Education, 70(3), 206–220. DOI: 10.2307/2673209

[46] Melamed, S., Ben-Avi, I., Lu, J., & Green, M. S. (1995). Objective and subjective work monotony: Effects on job satisfaction, psychological distress, and absenteeism in blue-collar workers. Journal of Applied Psychology, 80, 29–42. DOI: 10.1037/0021-9010.80.1.29 7706193

[47] Milevsky, A., Schlechter, M., Netter, S., & Keehn, D. (2007). Maternal and paternal parenting styles in adolescents: Associations with self-esteem, depression and life-satisfaction. Journal of Child and Family Studies, 16(1), 39–47. DOI: 10.1007/s10826-006-9066-5

[48] Morrison, D., Cordery, J., Girardi, A., & Payne, R. (2005). Job design, opportunities for skill utilization, and intrinsic job satisfaction. European Journal of Work and Organizational Psychology, 14(1), 59–79. DOI: 10.1080/13594320444000272

[49] Mortimer, J. T. (2003). Working and growing up in America. Cambridge, MA: Harvard University Press.

[50] Nes, R. B., Czajkowski, N. O., Røysamb, E., Ørstavik, R. E., Tambs, K., & Reichborn-Kjennerud, T. (2013). Major depression and life satisfaction: A population-based twin study. Journal of Affective Disorders, 144(1–2), 51–58. DOI: 10.1016/j.jad.2012.05.060 23021825 PMC3513516

[51] Neyt, B., Omey, E., Verhaest, D., & Baert, S. (2019). Does student work really affect educational outcomes? A review of the literature. Journal of Economic Surveys, 33, 896–921. DOI: 10.1111/joes.12301

[52] Nikolova, I., Van Ruysseveldt, J., De Witte, H., & Syroit, J. (2014). Work-based learning: Development and validation of a scale measuring the learning potential of the workplace (LPW). Journal of Vocational Behavior, 84, 1–10. DOI: 10.1016/j.jvb.2013.09.004

[53] Oberle, E., Schonert-Reichl, K. A., & Zumbo, B. D. (2011). Life satisfaction in early adolescence: Personal, neighborhood, school, family, and peer influences. Journal of Youth and Adolescence, 40(7), 889–901. DOI: 10.1007/s10964-010-9599-1 21042841

[54] O’Brien, G. E. (1980). The centrality of skill-utilization for job design In K. Duncan, M. Gruneberg, & D. Wallis (Eds.), Changes in working life (pp. 167–187). New York, NY: Wiley.

[55] Orth, U., Robins, R. W., & Roberts, B. W. (2008). Low self-esteem prospectively predicts depression in adolescence and young adulthood. Journal of Personality and Social Psychology, 95, 695–708. DOI: 10.1037/0022-3514.95.3.695 18729703

[56] Oswald, A. J., & Wu, S. (2010). Objective confirmation of subjective measures of human well-being: Evidence from the U.S.A. Science, 327(5965), 576–579. DOI: 10.1126/science.1180606 20019249

[57] Pavot, W., & Diener, E. (1993). Review of the Satifaction With Life Scale. Psychological Assessment, 5(2), 164–172. DOI: 10.1037/1040-3590.5.2.164

[58] Preacher, K. J., & Hayes, A. F. (2008). Asymptotic and resampling strategies for assessing and comparing indirect effects in multiple mediator models. Behavior Research Methods, 40(3), 879–891. DOI: 10.3758/BRM.40.3.879 18697684

[59] Proost, K., Van Ruysseveldt, J., & van Dijke, M. (2012). Coping with unmet expectations: Learning opportunities as a buffer against emotional exhaustion and turnover intentions. European Journal of Work and Organizational Psychology, 21(1), 7–27. DOI: 10.1080/1359432X.2010.526304

[60] Radloff, L. S. (1977). The CES-D scale: A self-report depression scale for research in the general population. Applied Psychological Measurement, 1(3), 385 DOI: 10.1177/014662167700100306

[61] Roberts, R. E., Andrews, J. A., Lewinsohn, P. M., & Hops, H. (1990). Assessment of depression in adolescents using the Center for Epidemiologic Studies Depression Scale. Psychological Assessment: A Journal of Consulting and Clinical Psychology, 2(2), 122–128. DOI: 10.1037/1040-3590.2.2.122

[62] Roberts, R. E., & Sobhan, M. (1992). Symptoms of depression in adolescence: A comparison of Anglo, African, and Hispanic Americans. Journal of Youth and Adolescence, 21(6), 639–651. DOI: 10.1007/BF01538736 24264167

[63] Ryan, R. M., & Deci, E. L. (2000a). Intrinsic and extrinsic motivations: Classic definitions and new directions. Contemporary Educational Psychology, 25, 54–67. DOI: 10.1006/ceps.1999.1020 10620381

[64] Ryan, R. M., & Deci, E. L. (2000b). Self-determination theory and the facilitation of intrinsic motivation, social development, and well-being. American Psychologist, 55(1), 68–78. DOI: 10.1037/0003-066X.55.1.68 11392867

[65] Salmelo-Aro, K., & Tuominen-Soini, H. (2010). Adolescents’ life satisfaction during the transition to post-comprehensive education: Antecedents and consequences. Journal of Happiness Studies, 11, 683–701. DOI: 10.1007/s10902-009-9156-3

[66] Shanahan, M. J., & Flaherty, B. P. (2001). Dynamic patterns of time use in adolescence. Child Development, 72(2), 385–401. DOI: 10.1111/1467-8624.00285 11333073

[67] Staff, J., & Schulenberg, J. E. (2010). Millennials and the world of work: Experiences in paid work during adolescence. Journal of Business and Psychology, 25(2), 247–255. DOI: 10.1007/s10869-010-9167-4 20495611 PMC2872249

[68] Steinberg, L., Fegley, S., & Dornbusch, S. M. (1993). Negative impact of part-time work on adolescent adjustment: Evidence from a longitudinal study. Developmental Psychology, 29(2), 171–180. DOI: 10.1037/0012-1649.29.2.171

[69] Stern, D., & Briggs, D. (2001). Does paid employment help or hinder performance in secondary school? Insights from US High School students. Journal of Education and Work, 14(3), 355–372. DOI: 10.1080/13639080120086148

[70] U.S. Bureau of Labor Statistics. (2006). College enrolment and work activity of 2005 high school graduates. Retrieved from http://www.bls.gov/news.release/hsgec.nr0.htm

[71] Valkenburg, P. M., Peter, J., & Schouten, A. (2006). Friend networking sites and their relationship to adolescents’ well-being and social self-esteem. Cyperpsychology & Behavior, 9(5), 584–590. DOI: 10.1089/cpb.2006.9.584 17034326

[72] van der Doef, M., & Maes, S. (1999). The Leiden Quality of Work Questionnaire: Its construction, factor structure, and psychometric qualities. Psychological Reports, 85(3), 954–962. DOI: 10.2466/pr0.1999.85.3.954 10672758

[73] Van der Stede, W. A. (2014). A manipulationist view of causality in cross-sectional survey research. Accounting, Organizations and Society, 39(7), 567–574. DOI: 10.1016/j.aos.2013.12.001

[74] Van Veldhoven, M., & Meijman, T. F. (1994). Measuring psychosocial workload by means of a questionnaire: Questionnaire on the experience and evaluation of work VBBA. Amsterdam, The Netherlands: NIA.

[75] Warren, J. R., & Cataldi, E. F. (2006). A historical perspective on high school students’ paid employment and its association with high school dropout. Sociological Forum, 21(1), 113–143. DOI: 10.1007/s11206-006-9005-7

[76] White, R. W. (1959). Motivation reconsidered: The concept of competence. Psychological Review, 66(5), 297–333. DOI: 10.1037/h0040934 13844397

[77] Wouters, S., De Fraine, B., Colpin, H., Van Damme, J., & Verschueren, K. (2012). The effect of track changes on the development of academic self-concept in high school: A dynamic test of the Big-Fish-Little-Pond effect. Journal of Educational Psychology, 104, 793–805. DOI: 10.1037/a0027732

[78] Young, J. E., Williamson, M. I., & Egan, T. G. (2016). Students’ reflections on the relationship between safe learning environments, learning challenge and positive experiences of learning in a simulated GP clinic. Advances in Health Sciences Education, 21, 63–77. DOI: 10.1007/s10459-015-9611-3 25952645

